# Rural providers’ attitudes toward integrating harm reduction strategies and PrEP prescribing into rural primary care settings in the US. Southeast and Midwest

**DOI:** 10.1186/s13722-025-00584-9

**Published:** 2025-09-12

**Authors:** Phillip L. Marotta, Miryam Biaid, Robert Heimer, Debbie Humphries, Katie Wang, Nithya Narayanan, Zach Lynch, Virginia McKay, Hilary Reno, Rachel Winograd, Dawn Goddard-Eckrich, Lindsey Filiatreau, Kristi Stringer, Kaileigh Backes, Patricia Cavazos-Rehg

**Affiliations:** 1 Brown School, Washington University, St. Louis, MO USA; 2 School of Public Health, Washington University, St. Louis, MO USA; 3 Yale School of Public Health, New Haven, CT USA; 4 Center for Methods in Implementation and Prevention Science, Yale School of Public Health, New Haven, CT USA; 5 Department of Social and Behavioral Sciences, Yale School of Public Health, New Haven, CT USA; 6 School of Medicine, Washington University, St. Louis, MO USA; 7 Department of Psychological Sciences, University of Missouri, St. Louis, MO USA; 8 Columbia University School of Social Work, New York, NY USA; 9 Department of Health and Human Performance, Middle Tennessee State University, Murfreesboro, TN USA

## Abstract

**Background:**

People with opioid use disorders (OUD) living in the South and Midwest are under-prescribed pre-exposure prophylaxis (PrEP) despite an increasing number of providers writing PrEP prescriptions in other regions of the United States. Greater research is needed into attitudes toward integrating harm reduction strategies into primary care and PrEP prescribing among prescribers working in rural primary care settings. The objective of this paper was to examine relationships between providers’ attitudes toward buprenorphine and methadone, comprehensive harm reduction (e.g., fentanyl test strips) and self-reported PrEP prescribing in the past year.

**Methods:**

Relationships were examined between attitudes toward buprenorphine and harm reduction services, and PrEP prescribing among 409 rural primary health care providers (PCPs) treating at least one person with OUD or HIV in several EHE priority states. A Qualtrics panel survey was administered to primary care providers residing in the U.S. South and Midwest and worked in a Federally Qualified Health Center, Rural Health Clinic or other HRSA-eligible health center. Chi-square tests were used to explore significant differences between PrEP prescribers and non-Prescribers on attitudes toward integrating MOUD, drug-related harm reduction into primary care.

**Results:**

Overall, 62.1% (*n* = 254) of the sample of providers reported writing at least one prescription for PrEP in the past year. Providers who believed that reforming buprenorphine waiver laws made their jobs easier or who expressed interest in integrating naloxone distribution and syringe exchange into primary care were more likely to write prescriptions for PrEP compared to providers who did not have these beliefs. Providers who were from larger facilities and who had specialty training in infectious diseases were more likely to write prescriptions for PrEP.

**Conclusions:**

Findings from this study suggest that providers who are more open to integrating harm reduction services into primary care are more likely to prescribe PrEP in the past year. PCPs with more positive attitudes toward naloxone, syringe exchange, and buprenorphine were more likely to prescribe PrEP in the past year. Combination interventions may be a promising avenue of reducing the harms of drug use including overdose and HIV infection among populations of people who use drugs.

## Introduction

Since first approved by the Food and Drug Administration (FDA) in 2012, antiretroviral drug combinations including emtricitabine and tenofovir used as preexposure prophylaxis (PrEP) have provided an effective strategy for preventing the acquisition of HIV [1, 2]. PrEP is now a widely accepted prevention strategy for the estimated 750,000 people who inject drugs (PWID) in the U.S [3–7]. The Ending the HIV Epidemic (EHE) initiative in the United States (US) aims to reduce the number of new HIV infections by 90% to fewer than 3000 annually by 2030 thus making HIV a rare disease [8, 9]. Relative to other regions of the U.S., people in the South and Midwest have less access to HIV-related healthcare, and that extends to the provision of PrEP [10, 11]. The syndemic of opioid use disorders (OUD), HIV, and hepatitis C virus (HCV) continues to worsen in the rural US imperiling the success of the US EHE Initiative that requires comprehensive harm reduction interventions to address the co-occurrence of these health problems [12–16]. Increasing access to MOUD improves HIV and HCV prevention and treatment outcomes [4, 17, 18]. However, only one in five people in the US with OUD who need treatment receive medication [19]. 

Comprehensive harm reduction includes scaling up adoption of PrEP alongside medication for opioid use disorders (MOUD), syringe exchange, naloxone distribution and fentanyl test strips in the rural South and Midwest [12–15]. MOUD includes the opioid agonists methadone and buprenorphine and the antagonist naltrexone. Buprenorphine is available as Suboxone^®^, a combination of buprenorphine and naloxone at a ratio of 4:1, Subutex^®^, which consists of only buprenorphine, and several prolonged released formulations [20] Elimination of the buprenorphine waiver requirements as part of the Consolidated Appropriations Act in 2023 unlocked new opportunities for the expansion and integration of treatment for OUD into primary care [21, 22]. Research has increasingly found that patients must travel extended distances to receive treatment, underscoring the need for research that examines primary care providers perspectives on providing MOUD and PrEP prescribing in primary care settings [23, 24]. Research is needed that examines the overlap of providers attitudes about eliminating the waiver buprenorphine waiver requirements, integrating MOUDs and harm reduction and PrEP prescribing to identify novel strategies that address the co-occurrence of HIV risk and substance use.

### Clinical addiction services and PrEP prescribing in rural primary care settings

The delivery of primary health care in rural areas occurs through Rural Health Clinics (RHCs), Federally Qualified Health Centers (FQHCs), and other health centers designated as eligible for Health Resource Services Administration (HRSA) funding because of being in shortage areas [25]. For a facility to be classified as a rural health clinic, it must be in a shortage area, not a facility primarily for psychiatric treatment, and provide preventative and primary care services [26, 27]. The EHE initiative came with investments by HRSA into FQHCs and RHCs located in the seven ending the HIV epidemic priority states of Alabama, Arkansas, Mississippi, Missouri, Oklahoma, Kentucky, and South Carolina [26]. 

Rural communities face unique challenges, including limited public transportation, geographic isolation, lags in the implementation of Medicaid expansion, and criminalization of syringe possession and service programs [28–32]. Prior studies have documented urban-rural disparities in the response to the OUD crisis, including higher rates of attrition and poorer rates of engagement in buprenorphine treatment among those with OUD in rural compared to urban areas [29, 31, 33–37]. Taken together, these issues underscore the need to expand the availability of services at primary care clinics that bundle OUD, PrEP, and other harm reduction strategies to address the syndenic and prevent the HIV transmission and overdose among people with OUD in rural areas.

### Integration of harm reduction services as a strategy of increasing access to combination interventions for HIV infection and overdose in rural primary care settings

Combination interventions package employ more than one strategy or harm reduction technique, (e.g. naloxone, buprenorphine, PrEP, syringe exchange, and drug checking kits) to co-target syndemic drivers of HIV infection, sexual health, and OUD. The syndemic perspective formulated by Merrill Singer investigates the social, behavioral, and biological factors that drive the co-occurrence of two or more epidemics [38, 39]. Syndemic control strategies are possible interventions that aim to reduce the co-occurrence of more than one of these chronic illnesses by addressing underlying drivers of multiple illnesses such as lack of access to MOUD, trauma-informed approaches, and discrimination and stigma [38, 40, 41]. By addressing syndemic drivers of poor health outcomes, it is possible to reduce both epidemics simultaneously. Many prior studies have emphasized the importance of advancing combination HIV prevention strategies to reduce HIV infections and drug related harms among people who inject drugs [42–45]. Despite these studies, research is lacking that examines combination interventions in the context of rural primary care providers.

### Attitudes toward integrating medication for opioid use disorders into rural primary care and PrEP prescribing settings

In recent years, there has been an increasing focus placed on identifying successful models for the integration of buprenorphine, and naltrexone into primary care settings [46, 47]. Primary care settings are a major venue for the identification and long-term management of OUD as a chronic disease [48]. The elimination of buprenorphine waiver requirements for physicians prescribing buprenorphine resulted in significantly more prescribers who could provide buprenorphine for people living in rural areas. Research has found that about a third of providers agreed that removing the buprenorphine waiver was a positive step in the right direction [21]. In addition to buprenorphine and naltrexone, there is a growing attention placed on the untapped potential of primary care providers to manage methadone treatment as a promising strategy of increasing the availability of treatments for OUD [49]. 

### Integration of drug-related harm reduction services into rural primary care settings

Despite the opportunity for systematic introduction of harm reduction interventions to address unmet comorbid HIV and drug-related needs simultaneously in rural communities, syringe services, naloxone distribution, and drug checking test strips and kits are yet to be integrated into most primary care settings [50, 51]. Primary care is recognized as an underserved setting for the distribution of naloxone [52]. Providing naloxone in primary care settings has been found acceptable and feasible to primary care provider (PCPs), and especially in circumstances when co-prescribing opiates analgesics, buprenorphine and polypharmacy [51, 53, 54]. 

### Gaps in the existing research

Primary care clinicians are on the frontlines of efforts to increase coverage of PrEP, MOUD, and other harm reduction strategies among people who use drugs, yet gaps exist in the implementation of harm reduction strategies in primary care settings [55]. Researchers have called for integrated MOUD and HIV treatment within primary care settings, particularly for PWID [13, 56–59]. The integration of HIV prevention and OUD treatment into primary care settings could significantly advance population level approaches to addressing the drivers of the syndemic of opioid use and HIV infection among people living in rural areas. However, few studies have focused on primary care providers’ attitudes toward PrEP and other comprehensive harm reduction strategies in rural areas of the Midwest and U.S Southeast where rates of HIV and overdose remain high [60]. The aim of this study was to carry out an exploratory analysis investigating the primary care providers’ attitudes toward comprehensive harm reduction and PrEP prescribing and how these attitudes overlapped. The decision was made to focus recruitment inclusion of primary care providers already working with populations of people with OUD and HIV and therefore engaging with populations at greatest risk of experiencing the harms of drug use.

## Methods

### Study design

This exploratory, cross-sectional study was conducted to compare self-reported PrEP prescribers to those who did not write prescriptions for PrEP in the past year regarding attitudes toward buprenorphine and methadone and an interest in integrating drug-related harm reduction services into primary care. A survey was administered to participants who were eligible if they reported working in primary care settings in rural geographic areas within one of the EHE priority states.

Participants were identified and screened using a Qualtrics research panel of primary care providers from six EHE priority states – Alabama, Arkansas, Kentucky, Mississippi, Missouri, and Oklahoma. A non-representative sample of 409 providers were selected from rural counties using the HRSA definition of rural [26]. We chose to use a Qualtrics panel since the service builds and maintains panels from multiple sources of respondents and are used in numerous prior studies [60–62]. Panels are assembled and maintained by Qualtrics based on identifying medical care providers who completed previous surveys, responded to advertisements and publicly available data, and agreed to complete surveys in exchange for compensation.

For this study, panel members were eligible if they: (a) worked in primary care settings, (b) treated at least one person with HIV or opioid use disorder, (c) legally allowed to prescribe medications, (d) worked in a county within one of the seven Ending the HIV epidemic states and (e) currently employed by a FQHC, RHC or HRSA eligible clinic. Data collection occurred between May 23rd, 2023, and June 12th, 2023. Individuals providing incomplete responses to survey questions were dropped from the data analysis. Convenience sampling was used and therefore sample recruitment was not designed to be representative of the larger population of providers. Measures were not taken to ensure the sample of providers were representative of providers in the community on characteristics such as race, age, gender and other factors.

### Ethics

#### Informed consent

was obtained electronically followed by an online self-administered anonymous survey that took on average approximately 45 min to complete. No identifiable information, including IP addresses, was collected ensuring respondent privacy. The Washington University and Yale University Institutional Review Boards approved this study.

### Measures

#### Survey development

Prior to administering the survey, input and feedback was requested from state agencies and associations representing health care professionals in Missouri. Feedback on the survey solicited advice on question comprehension, potential response bias, and question fatigue. Survey questions for this study were taken from prior research but did not come from validated scales.

#### Pre-Exposure prophylaxis (PrEP) prescribing

Participants were asked to indicate how many patients they prescribed PrEP to within the past year and month; response options include none, 1–10, 11–20, 21–30 and > 30. A dichotomous variable was created indicating any PrEP prescribing in the past year (any, none).

#### Independent variables

Characteristics of providers and facilities included age, medical credentials (MD/DO, psychiatry, and physician assistants), specialty training (psychiatry, internal medicine, infectious disease, primary care and family medicine and OB/GYN), sex (male, female), gender (female, male), race (white, Black, American Indian, Alaska Native, Pacific Islander), Hispanic ethnicity, age (years), number of patients with substance use disorders, percentage of patients with opioid, alcohol, stimulant use disorders and who injected drugs (none, > 0-<25%, 25-<50%, 50-<75%, 75–100%). Characteristics of facilities included number of patients treated by the provider’s facility each year (1-499, 500–999; ≥1000), health center funding status (FQHC, RHC, or HRSA-eligible centers), having patients on public assistance (yes, no), and the state where the facility is situated.

#### Treatment and drug-related harm reduction services

Medication for opioid use disorder (MOUD) provision included prescribing Suboxone^®^, oral buprenorphine without naloxone (Subutex^®^), or the one injectable form of buprenorphine available when the study started (Sublocade^®^). Additional questions on provision of MOUD included whether the provider had been waivered prior to elimination of the X waiver (yes, no), total number of patients on buprenorphine in the past year (none, 1–19, 20–29, ≥ 30).

### Attitudes toward integrating MOUD, overdose prevention, and infectious disease treatment and prevention into primary care and providing services to people involved in the criminal legal system

**Buprenorphine**. Providers were asked to report the extent to which they agreed that reforming buprenorphine waiver laws made their jobs easier, the extent that reforming buprenorphine waiver laws has increased prescribing of buprenorphine, and their interest in providing buprenorphine treatment at their facility. **Methadone**. Providers were asked how interested they were in integrating methadone into their current practice and belief that primary care providers should be able to prescribe methadone. **Naloxone distribution.** Providers were asked how interested they were in integrating naloxone distribution into primary care and the extent to which they agree with the statement that providing naloxone over the counter would make it easier to get naloxone into the hands of people who need it most. **Harm reduction services.** Providers were asked to indicate their interest in integrating syringe service and fentanyl test strips into primary care and their attitudes toward specific harm reduction services.

### Statistical analyses

Descriptive statistics and bivariate analyses were performed using chi-square tests to determine significant differences on all the characteristics of providers, attitudes toward MOUD, and integration of harm reduction into primary care services and PrEP prescribing [61–63]. Tables are presented with the prevalence of each characteristic for 409 eligible providers who completed the survey.

## Results

### Descriptive characteristics of providers working in primary care settings

Of the 409 eligible, 69.4% (*n* = 254) had prescribed PrEP to at least one person in the year prior. Table 1 presents frequencies and proportions of providers’ sociodemographic and clinical practice characteristics and differences by past year PrEP prescribing. Most reported having a medical degree (MD/DO) (60.9%, *n* = 249) and the remaining sample evenly divided between nurse practitioners (NPs) (19.8%, *n* = 81) and physician assistants (PAs) (19.3%, *n* = 79). Nearly all reported having specialty training in primary care (94.1%, *n* = 385), more than two thirds reported having specialty training in infectious diseases (66.5%, *n* = 272) and nearly half reported specialty training in internal medicine (48.7%, *n* = 199) or family medicine (46.5%, *n* = 190). Approximately 60% was male (*n* = 242. They were overwhelmingly White (86.8%, *n* = 355) with the proportion Black/African American (12.5%, *n* = 51) accounting for 12% of the sample. More than half worked in Rural Health Clinics (RHCs) (58.2%, *n* = 238) compared to 31.3% (*n* = 128) in FQHCs and 10.5% (*n* = 43) in HRSA eligible health centers. Participants from Kentucky (24.5%, *n* = 100) and Oklahoma (24.5%, *n* = 100) accounted for almost half of the sample, followed by Missouri (18.6%, *n* = 76), Mississippi (17.9%, *n* = 73), Arkansas (13.5%, *n* = 55), and Alabama (1.2%, *n* = 5).

**Table 1:** Descriptive statistics and tests of differences between characteristics of providers and past year PrEP prescribing among rural primary care providers in 6 of the 7 EHE States (*n* = 409)

	Past Year PrEP Prescribing				
	Overall	None (*n* = 155) %(n)	Any (*n* = 254) %(n)	χ2	p-value
**Characteristics of providers**					
Medical Credentials					
MD/DO	60.9(249)	49.0(76)	68.1(173)	14.7	**< 0.001**
NP	19.8(81)	29.7(46)	13.8(35)	15.4	**< 0.001**
PA	19.3(79)	21.3(33)	18.1(46)	0.625	0.429
Sex assigned at birth				3.3	0.071
Female	40.8(167)	46.5(72)	37.4(95)		
Male	59.2(242)	53.6(83)	62.6(159)		
Race					
Other (Amer. Indian or Alaska Native, Asian, N. Hawaiian, Pacific Islander)	0.73(3)	1.3(2)	0.4(1)	-	-
Black or African American	12.5(51)	11.0(17)	13.4(34)	0.516	0.473
White	86.8(355)	88.4(137)	85.8(218)	0.55	0.458
Hispanic	3.7(15)	1.9(3)	4.7(12)	-	-
Age	48.9(1.8)	50.1(1.2)	48.2(1.1)	0.56	0.461
**Substance use disorders**					
How many of your patients have diagnoses of substance use disorders				15.3	**0.002**
1–10	64.1(262)	54.2(84)	70.1(178)		
11–20	33.5(137)	40.7(63)	29.1(74)		
21–30	2.2(9)	4.5(7)	0.7(2)		
31–40	0.2(1)	0.7(1)	0(0)		
What percentage of those patients have opioid use disorders?				5.2	0.158
0	4.4(18)	7.1(11)	2.8(7)		
< 25%	67.0(274)	66.5(103)	67.3(171)		
25–49%	23.5(96)	20.7(32)	25.2(64)		
~ 50%	5.1(21)	5.8(9)	4.7(12)		
How many of your patients have injected drugs?				7.5	0.184
None					
0 to < 25%	55.3(226)	60.7(94)	52.0(132)		
25–49%	28.1(115)	28.4(44)	28.0(71)		
~ 50%	6.5(10)	9.5(24)	8.3(34)		
51–74%	2.9(12)	1.9(3)	3.5(9)		
> 74%	1.0(4)	0(0)	1.5(4)		
**Facility level factors**					
How many patients does your facility treat?				32.9	**< 0.001**
1-499	65.5(268)	82.6(128)	55.1(140)		
500–999	21.0(86)	12.3(19)	26.4(67)		
1000+	13.5(55)	5.2(8)	18.5(47)		
Health Center Funding Status				12.1	**< 0.001**
Federally Qualified Health Center (FQHC)	31.3(128)	25.8(40)	34.7(88)		
Rural Health Center	58.2(238)	68.4(106)	52.0(132)		
HRSA Eligible Health Center	10.5(43)	5.8(9)	13.4(34)		
**Ending the HIV Epidemic States**					
Alabama	1.2(5)	0(0.0)	2.0(5)	ND	**ND**
Arkansas	13.5(55)	11.0(17)	15.0(38)		
Kentucky	24.5(100)	20.0(31)	27.2(69)		
Mississippi	17.9(73)	16.1(25)	18.9(48)		
Missouri	18.6(76)	25.8(40)	14.2(36)		
Oklahoma	24.5(100)	27.1(42)	22.8(58)		

The most prescribed buprenorphine medication, Suboxone^®^, was prescribed by 53.3% (*n* = 218) of the providers. Sublingual buprenorphine was prescribed by 31.5% (*n* = 129) and injectable Sublocade^®^ was prescribed by 20.8% (*n* = 85). More than 30% of providers reported treating no patients with buprenorphine (31.1%, *n* = 127), slightly fewer treated between 1 and 19 (29.8%, *n* = 122) and 20–29 (27.4%, *n* = 112), and 11.7% (*n* = 48) reported treating 30 or more. Most of the sample reported agreeing or strongly agreeing that reforming buprenorphine waiver laws made their job easier (65.0%, *n* = 266) and that a plan for tapering should be discussed with the patient prior to initiating buprenorphine (77.5%, *n* = 317).

### Significant differences between PrEP prescribers and non-prescribers

#### Buprenorphine

Among 254 PrEP prescribers, 205 (81.0%) reported being buprenorphine waivered providers prior to the elimination of the DATA waiver requirements whereas among 155 non-prescribers, only 83 (53.6%) had been waivered (χ2 = 35.0, *p* < .001). Past year PrEP prescribers were more likely to provide Suboxone^®^ (61.4%, *n* = 156 vs. 40.0%, *n* = 62, χ2 = 17.7, *p* < .001), Subutex (37.4%, *n* = 95 vs. 21.9%, *n* = 34, χ2 = 10.7, *p* < .001) and Sublocade (24.0%, *n* = 61 vs. 15.5%, *n* = 24, χ2 = 4.3, *p* = .039) as treatment options compared to providers who did not write prescriptions for PrEP. Past year PrEP prescribers reported writing prescriptions for significantly more patients to receive buprenorphine compared to non-past year PrEP prescribers (χ2 = 40.4, *p* < .001).

Significantly more PrEP prescribers were physicians compared to PrEP non-prescribers (49.0%, *n* = 76 vs. 68.1%, *n* = 173 χ2 = 14.7, *p* < .001). Fewer nurse practitioners (NPs) were PrEP prescribers compared to non-prescribers (13.8% *n* = 35 vs. 29.7%, *n* = 46, χ2 = 15.4). The remainder of the sample were physician assistants (*n* = 79, 19.3%). There was no significant difference observed between PrEP prescribers compared to non-prescribers ((21.3% (*n* = 33) vs. 18.1% (*n* = 46)). Significantly more PrEP prescribers reported specialty training in infectious diseases (80.3%, *n* = 204 vs. 43.9% *n* = 68, χ2 = 57.4, *p* < .001) and primary care (96.1% *n* = 244 vs. 91.0 *n* = 141, χ2 = 4.5, *p* = .033) compared to non-prescribers.

#### Hepatitis C (HCV) treatment

Past year PrEP prescribers reported having significantly more patients with HCV and ordered an HCV test more recently compared to non-providers (χ2 = 72.4, *p* < .001). Moreover, significantly more past year PrEP prescribers reported ordering an HCV in the past month or week compared to non-prescribers (χ2 = 77.8, *p* < .001).

### Attitudes toward integrating MOUD into primary care settings

#### Buprenorphine

Table 2 presents frequencies and tests of statistical differences between attitudes toward integrating MOUD into primary care settings and past year PrEP prescribing. A greater proportion of past year PrEP providers agreed or strongly agreed with statements that reforming buprenorphine requirements made their jobs easier compared with non-prescribers (77.9%, *n* = 198 vs. 43.9%, *n* = 68; χ2 = 40.7, < 0.001). They were significantly more interested in providing buprenorphine treatment to patients at their facility compared to non-prescribers (87.8%, *n* = 223 vs. 60.7%, *n* = 94; χ2 = 40.7, *p* < .001) and more likely to report increased prescribing of buprenorphine (78.0%, *n* = 198 vs. 43.9%, *n* = 68; χ2 = 49.2, < 0.001) since removal of the waiver.

**Table 2:** Descriptive statistics and tests of significant differences between services for the treatment of substance use disorders, and past year PrEP prescribing among rural primary care providers (*n* = 409)

			Past Year PrEP Prescribing			
Provision of MOUD		Overall	None (*n* = 155) %(*n*)	Any (*n* = 254) %(*n*)	χ2	*p*-value
Buprenorphine provision						
	Suboxone	53.3(218)	40.0(62)	61.4(156)	17.7	**< 0.001**
	Subutex	31.5(129)	21.9(34)	37.4(95)	10.7	**< 0.001**
	Injectable Sublocade	20.8(85)	15.5(24)	24.0(61)	4.3	**0.039**
Prior to elimination of X-waiver were you a waivered provider?		70.6(288)	53.6(83)	81.0(205)	35	**< 0.001**
Total number of patients on buprenorphine in the past year.					40.4	**< 0.001**
	None	31.1(127)	47.7(74)	20.9(53)		
	1–19	29.8(122)	22.0(34)	34.7(88)		
	20–29	27.4(112)	16.1(25)	34.3(87)		
	30+	11.7(48)	14.2(22)	10.2(26)		
**Overdose prevention**						
	Offer naloxone to people with a diagnosed/suspected OUD? (Sometimes or often)	69.7(285)	58.7(91)	76.4(194)		**< 0.001**
Number of patients with hepatitis C					72.4	**< 0.001**
	None	17.6(72)	38.1(59)	5.1(13)		
	1–10	71.6(293)	54.2(84)	82.3(209)		
	11–20	10.5(43)	7.7(12)	12.2(31)		
	21–30	0.2(1)	0(0)	0.39(1)		
Last time ordered Hepatitis C test					77.8	**< 0.001**
	Never	18.9(77)	40.0(62)	5.9(15)		
	Within a month	35.5(145)	21.3(33)	44.1(112)		
	Within a week	45.0(184)	37.4(58)	49.6(126)		
	Within a day	0.7(3)	1.3(2)	0.4(1)		

#### Methadone

A greater proportion of recent PrEP prescribers agreed that primary care providers should be able to prescribe methadone compared to non-prescribers (28.7%, *n* = 73 vs. 14.2%, *n* = 22; χ2 = 11.4, *p* < .001) and were interested in integrating methadone into primary care was significantly greater than among non-providers (77.6%, *n* = 297, vs. 68.4%, *n* = 106, χ2 = 4.2, *p* = .040).

### Attitudes toward integrating comprehensive harm reduction services

Table 3 presents frequencies and tests of differences between attitudes toward integrating comprehensive harm reduction services and past year PrEP prescribing. Significantly more past year PrEP prescribers reported being interested or very interested in integrating fentanyl test strip distribution (87.4%, *n* = 222 vs. 69.7%, *n* = 108; χ2 = 19.4, *p* < .001), and naloxone distribution services (85.4%, *n* = 217; vs. 54.2%, *n* = 84; χ2 = 48.3 *p* < .001) into primary care. Significantly more past year PrEP prescribers than non-prescribers reported being interested in integrating syringe exchange (93.7% *n* = 238 vs. 73.6% *n* = 114; χ2 = 32.6, *p* < .001).

**Table 3:** Descriptive statistics and tests of significant differences between attitudes toward MOUD, interest in harm reduction services, and PrEP prescribing among rural primary care providers (*n* = 409)

		Past Year PrEP Prescribing				
		**Overall**	**None** (*n* = 155)	**Any** (*n* = 254)		**p-value**
**Attitudes toward integrating MOUD into primary care**		**%(n)**	%(n)	%(n)	**χ2**	
**Buprenorphine**						
	Reforming buprenorphine waiver requirements has made my job easier.	65.0(266)	43.9(68)	77.9(198)	49.1	**< 0.001**
	Reforming buprenorphine waiver laws has increased prescribing of buprenorphine at my facility.	65.0(266)	43.9(68)	78.0(198)	49.2	**< 0.001**
	How interested are you in providing buprenorphine treatment to patients at your facility?	77.5(317)	60.7(94)	87.8(223)	40.7	**< 0.001**
**Methadone**						
	How interested are you in integrating methadone into your current practice?	74.1(303)	68.4(106)	77.6(297)	4.2	**0.04**
	I believe that primary care providers should be able to prescribe methadone	23.2(95)	14.2(22)	28.7(73)	11.4	**< 0.001**
**Attitudes toward overdose prevention**						
	How interested are you in integrating Naloxone distribution into primary care?	73.6(301)	54.2(84)	85.4(217)	48.3	**< 0.001**
	How interested are you in providing fentanyl test strip services into primary care	80.7(330)	69.7(108)	87.4(222)	19.4	**< 0.001**
**Interest in integrating drug-related infectious disease transmission and prevention into primary care**						
	How interested are you in integrating syringe exchange services into primary care	86.1(352)	73.6(114)	93.7(238)	32.6	**< 0.001**
	How interested are you in integrating Hepatitis C treatment into primary care	83.1(340)	63.9(99)	94.9(241)	66.0	**< 0.001**
**Attitudes toward the criminal legal system**						
	People who are reentering the community from periods of incarceration deserve access to free sexual health services at FQHCs and other health centers.	55.5(227)	44.5(69)	62.2(158)	12.2	**< 0.001**

## Discussion

Findings from this study contribute new insights into relationships between providers’ personal characteristics, facility characteristics, interest in integrating harm reduction services into primary care as a function of past year PrEP prescribing. Results suggest that advancing opportunities for PrEP prescribers to integrate harm reduction practices and MOUD in the context of HIV prevention in primary care settings could significantly address the syndemic of opioid use and HIV infection in rural settings where rates of HIV continue to expand. These results are consistent with existing literature in settings other than rural areas suggesting that, regardless of locale, providers who engaged in HIV prevention by prescribing PrEP may be more open to integrating other types of comprehensive harm reduction strategies [64–66]. These prescribers were more likely to work in larger facilities, those that were FQHCs, and were more likely to have an MD or DO degree compared to non-prescribers. These findings are consistent with existing research suggesting that FQHCs are locations where people who use substances may benefit from greater access to PrEP that is integrated with other comprehensive harm reduction services such as syringe services [67, 68]. Providers who reported having a greater proportion of patients with alcohol use disorders and stimulant use disorders were more likely to write prescriptions for PrEP. The findings presented in this paper emphasize the overlap between positive attitudes toward PrEP and other harm reduction interventions among prescribers working in rural primary care settings in the EHE priority states. These findings is also consistent with research that suggests prescribers who are writing prescriptions for MOUD in other settings are open to offering PrEP prescriptions at the same time [69, 70]. The study is timely considering the persistent high rates of opioid overdose and HIV in many areas of the rural Midwest and the South, and it points to the critical need in the field for models of integration of prevention strategies for OUD and HIV in rural primary care settings, particularly rural health clinics and FQHCs. The work in this study filled a major gap in prior research by extending this work to providers working in rural settings in several states within the EHE Initiative.

Several clinical practice implications for prescribers working with people with OUD in rural primary care settings such as FQHCs emerge from this study. While prior studies have primarily focused on provider attitudes toward PrEP implementation as drivers of PrEP prescribing without including other harm reduction services [71–74], this study is among the first to focus on relationships of interests in integrating syringe exchange and fentanyl test strip distribution into primary care, attitudes toward distribution of naloxone in primary care with the likelihood that providers reported writing prescriptions for PrEP in the past year. The study also indicates that there remain considerable barriers to the expansion of integrated service provision based on the attitudes of a substantial minority of care providers themselves. Since a growing body of research suggesting that combination interventions are more promising to prevent HIV infection and opioid overdose than addressing each health problem and drug-related harm separately [42–44, 75, 76], our findings suggest that academic detailing for active practitioners that emphasizes a syndemic approach to patient care could have a large impact on both problematic opioid use and HIV transmission.

### Limitations

This study has several limitations that will provide potentially fruitful avenues of future research. First, this study is cross-sectional, and thus causal inference is not possible from estimates presented in this study. The findings presented in this paper represent an exploratory analysis of the intersection between providers’ attitudes toward integrating buprenorphine post-waiver elimination, harm reduction and overdose prevention services and providers’ attitudes towards PrEP. Providers in this study were not asked to verify any of our responses using chart review or any kind of external checking of accuracy of patient caseloads or patient population demographics. This study relies on the estimation by participants of the composition of their patient population and their respective facilities. There may be some bias that is introduced into the study because of desirability or memory related errors in biases. Future research that blends self-report with verification through chart review could improve the accuracy of findings reported in this study.

The focus of this analysis was on PrEP prescribing relative to other factors in the HIV/OUD syndemic. It examined the overlap between attitudes toward integrating harm reduction services, prescribing buprenorphine, and PrEP prescribing. It remains to be assessed to what extent providers’ attitudes toward PrEP and comprehensive harm reduction are a function of the policies and culture of their clinic workplace, the impact of subspecialty training in areas such as infectious diseases, psychiatry, and family medicine, or structural or cultural changes in the communities where their clinics are located.

Another major limitation of this study is the number of providers who prescribed PrEP explicitly for people with OUD was not measured. That is, some prescribers might choose to prescribe PrEP only to some classes of patients while failing to prescribe it to others. Future research must examine attitudes among providers working in primary care settings.

Another limitation is that one inclusion criterion was experience treating patients with HIV or OUD; therefore, it is not reflective of PrEP prescribing patterns of primary care providers in general. The sample may be generalizable to primary care providers treating at least one person with OUD or HIV, but even if that, only to any rural primary providers. Another limitation worth noting is that providers were from small facilities; most had fewer than 1000 patients annually. This study does not reflect the population of providers from larger facilities. More broadly, this study was not intended to be nationally representative of the general population of primary care providers.

Finally, ours was a cross-sectional study. Longitudinal is needed to examine how changes in the underlying structure of professional and cultural values shape providers’ decisions to adopt evidence-based harm reduction strategies. Future qualitative research should build upon the work started by this study, collecting data that uses scenarios and vignettes to measure flexibilities in providers attitudes and judgement around implementing evidence-based harm reduction strategies.

## Conclusion

Findings from this study are intended to inform potential interventions directed to providers to help increase positive attitudes toward the implemention of harm reduction measures in their clinics. Providers who prescribe PrEP appear to be more open to integrating buprenorphine compared to non-prescribers. The provision of buprenorphine and PrEP in primary care settings seems to open primary care providers willingness to deliver multiple HIV and overdose prevention services in a single location. Expanding buprenorphine and PrEP prescribing into rural primary care practices may be an important step in expanding syndemic responses that advance the prevention of overdose and HIV epidemics among people with OUD in the US.

## Data Availability

The datasets analyzed during the current study are available from the corresponding author on reasonable request.
